# Alkyl-Resorcinol Derivatives as Inhibitors of GDP-Mannose Pyrophosphorylase with Antileishmanial Activities

**DOI:** 10.3390/molecules26061551

**Published:** 2021-03-11

**Authors:** Hélène Levaique, Olivier Pamlard, Cécile Apel, Jérôme Bignon, Margaux Arriola, Robin Kuhner, Khalijah Awang, Philippe M. Loiseau, Marc Litaudon, Sébastien Pomel

**Affiliations:** 1 CNRS, Institut de Chimie des Substances Naturelles, Université Paris-Saclay, UPR 2301, 91198 Gif-sur-Yvette, France; helene.levaique@cnrs.fr (H.L.); olivier.pamlard@cnrs.fr (O.P.); cecile.apel@cnrs.fr (C.A.); jerome.bignon@cnrs.fr (J.B.); marc.litaudon@cnrs.fr (M.L.); 2 CNRS, BioCIS, Université Paris-Saclay, 92290 Châtenay-Malabry, France; margaux.arriola@hotmail.fr (M.A.); robin.kuhner@gmail.com (R.K.); philippe.loiseau@universite-paris-saclay.fr (P.M.L.); 3 Department of Chemistry, Faculty of Science, University of Malaya, 50603 Kuala Lumpur, Malaysia; khalijah@um.edu.my

**Keywords:** *Leishmania*, therapeutic target, GDP-Mannose pyrophosphorylase, natural products, alkyl-resorcinol

## Abstract

Leishmaniasis is a vector-borne disease caused by the protozoan parasite *Leishmania* found in tropical and sub-tropical areas, affecting 12 million people around the world. Only few treatments are available against this disease and all of them present issues of toxicity and/or resistance. In this context, the development of new antileishmanial drugs specifically directed against a therapeutic target appears to be a promising strategy. The GDP-Mannose Pyrophosphorylase (GDP-MP) has been previously shown to be an attractive therapeutic target in *Leishmania*. In this study, a chemical library of 5000 compounds was screened on both *L. infantum* (*Li*GDP-MP) and human (*h*GDP-MP) GDP-MPs. From this screening, oncostemonol D was found to be active on both GDP-MPs at the micromolar level. Ten alkyl-resorcinol derivatives, of which oncostemonols E and J (**2** and **3**) were described for the first time from nature, were then evaluated on both enzymes as well as on *L. infantum* axenic and intramacrophage amastigotes. From this evaluation, compounds **1** and **3** inhibited both GDP-MPs at the micromolar level, and compound **9** displayed a three-times lower IC_50_ on *Li*GDP-MP, at 11 µM, than on *h*GDP-MP. As they displayed mild activities on the parasite, these compounds need to be further pharmacomodulated in order to improve their affinity and specificity to the target as well as their antileishmanial activity.

## 1. Introduction

Leishmaniases are a complex of neglected tropical diseases caused by the protozoan parasite from the genus *Leishmania* sp. and transmitted by an insect vector, the phlebotomine sandfly. Three main forms of clinical manifestations can be observed in leishmaniases depending on the *Leishmania* species considered: (i) the cutaneous form causing skin sores, which are often self-healing, provoked by *L. major* in North Africa or *L. mexicana* in South America for example, (ii) the muco-cutaneous form, which is highly disfiguring and is caused by *L. braziliensis* in South America, and (iii) the visceral form, which is lethal without treatment and is caused by *L. infantum* in the Mediterranean Basin or *L. donovani* in East Africa or in India for example [1]. Currently, more than 1 billion people are threatened by this parasitic disease worldwide, with 30,000 and 1 million new cases of visceral and cutaneous leishmaniasis, respectively [2]. During its life cycle, the parasite is characterized by two forms: a mobile flagellated and elongated promastigote form in the sandfly and a round, aflagellated and intracellular amastigote form in mammal hosts [3]. Only few treatments, including antimoniates, miltefosine, paromomycin and liposomal amphotericin B, are currently available against leishmaniases and present several issues of toxicity, emergence of resistance, and cost. The development of new antileishmanial agents is a priority in order to circumvent these treatment limitations. In particular, the identification of new compounds specifically directed against a therapeutic target in the parasite could be a promising approach.

The surface of *Leishmania* is composed of many mannose-containing glycoconjugates, such as lipophosphoglycans, proteophosphoglycans, glycosylinositolphospholipids, or glycosylphosphatidylinositol, which are essential for host cell recognition [4,5]. The mannosylation pathway requires the conversion of mannose into a nucleotide sugar, GDP-mannose, which is further used as a substrate by mannosyltransferases to transfer mannose on growing glycoconjugates. In this pathway, the formation of GDP-mannose is catalyzed by the GDP-Mannose Pyrophosphorylase (GDP-MP) from the substrates mannose-1-phosphate and GTP [6]. A knockout in *L. mexicana* demonstrated that GDP-MP is essential for amastigote survival both in vitro and in vivo [7,8]. Moreover, from molecular modelling studies, several differences have been evidenced in the catalytic site of leishmanial GDP-MP compared to the human counterpart, allowing the development of inhibitors specifically directed against the leishmanial enzyme [9,10]. Therefore, GDP-MP constitutes an interesting therapeutic target for the development of new specific antileishmanial agents. Indeed, two independent studies identified GDP-MP inhibitors with in vitro antileishmanial activities: one from a high-throughput screening of a library of small molecules with IC_50_ at the submicromolar range on the enzyme and at 21.9 µM on the parasite (compond−**100**) [11], and one from the screening of 100 compounds rationally designed from GDP-MP molecular models with a K*_i_* at 7 µM on the enzyme and an IC_50_ at the submicromolar level on the parasite (compound−**99**) [12].

In order to increase the diversity of leishmanial GDP-MP inhibitors, we screened a library of 5000 natural products on both recombinant leishmanial (*Li*GDP-MP from *L. infantum*) and human (*h*GDP-MP) in the present study. These leishmanial GDP-MP inhibitors were further analyzed for their in vitro antileishmanial activity.

## 2. Results and Discussion

**Figure 1:**
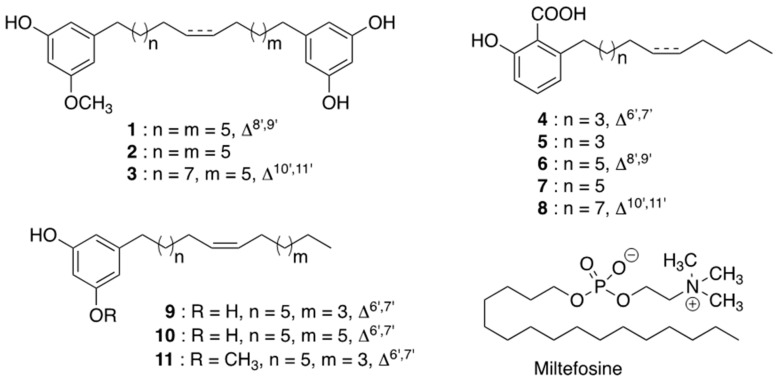
Structures of compounds **1**–**11.**

Following a screening of an in-house library of 5000 natural compounds on both leishmanial and human GDP-MP, oncostemonol D (**1**), which had been isolated from *Virotia francii* in a previous phytochemical study, was found to be active on both *Li*GDP-MP and *h*GDP-MP with similar IC_50_ at the micromolar range (Table 1). Moreover, the IC_50_ of this compound on both axenic and intramacrophage amastigotes was above 50 µM, with a CC_50_ at 34.4 µM, therefore giving a low selectivity index (SI < 1). These results prompted us to evaluate the efficacy of other structurally related compounds on both enzymes, *Li*GDP-MP and *h*GDP-MP, and on *L. infantum* amastigotes *in vitro*. Compounds **1–3** and **9–11** were isolated from the New Caledonian species *Virotia francii* (Proteaceae) and *Tapeinosperma schlechteri* (Primulaceae), respectively (Figure 1), while compounds **4–8** were isolated from the Malaysian species *Knema hookeriana* (Myristicaceae; Figure 1). Among these, only compounds **2** and **3**, oncostemonol I and oncostemonol J, respectively, presented also a resorcinol cycle at one end of the alkyl chain and a resorcinol monomethyl ether at the other end, as in **1**. However, unlike **1**, compounds **2** and **3** either lack the unsaturation at the C8 position (**2**; Figure 1), or present a slighter longer alkyl chain (**3**; Figure 1). Similar IC_50s_ were obtained on both *Li*GDP-MP and *h*GDP-MP, between 5.9 µM and 13.7 µM for compound **2**, and between 2.3 µM and 1.9 µM for compound **3**, showing a low specificity of action of these compounds on the leishmanial target. Moreover, oncostemonol J (**3**) displayed a similar antileishmanial activity to oncostemonol D (**1**), with an IC_50_ above 25 µM on both axenic and intramacrophage amastigotes (Table 1).

All the other compounds from the series presented either a resorcinol or a resorcinol monomethyl ether (**9–11**), or a salicylic cycle derivative (**4–8**), at only one end of the linear aliphatic chain (Figure 1). Compounds **4–8** are therefore anacardic acids. Among the anacardic acids, compounds **7** and **8** were the most active compounds on the leishmanial GDP-MP with IC_50_ at 16.9 µM and 9.8 µM, respectively (Table 1). Moreover, compound **8** displayed a similar IC_50_ on the human enzyme, while compound **7** was approximately two times less active on *h*GDP-MP showing a mild specificity of action of this latter compound on *Li*GDP-MP. Unfortunately, none of the anacardic acids displayed an activity on axenic amastigotes showing an absence of target inhibition within the parasite, despite an interesting activity on the leishmanial purified enzyme. Nevertheless, compound **6** displayed a mild antileishmanial activity on intramacrophage parasites, with an absence of cytotoxicity, giving therefore a potential interesting SI above 2.3. As this compound does not inhibit axenic amastigotes, it would not act directly on the parasite, but it would rather inhibit parasite growth via the host cell. This host-directed mechanism of action has also been described for other compounds [13,14,15].

Among the other resorcinol derivatives, compounds **9** and **10** have an unsaturated alkyl chain which only differs in the number of carbons: 15 for compound **9** and 17 for compound **10** (Figure 1). These compounds displayed interesting IC_50s_ of 11.0 µM and 14.1 µM, respectively, on the leishmanial GDP-MP (Table 1). Moreover, both compounds displayed smaller activities on *h*GDP-MP, with IC_50_ at 31.1 µM and 21.9 µM for **9** and **10**, respectively. These data show that both compounds would act with a slightly higher specificity on *Li*GDP-MP than on *h*GDP-MP. However, they displayed only a mild activity on axenic amastigotes and no significant inhibition of intramacrophage parasites was observed. These results show that the length of alkyl chain in these alkyl-mono-resorcinol does not have an effect on their antileishmanial activity.

Additionally, compound **11** has no activity on the leishmanial target (Table 1). This compound has the same structure as **9** except that it presents a resorcinol monomethyl ether cycle instead of a resorcinol cycle (Figure 1). These results show the importance of the resorcinol group in the alkyl-mono-resorcinol compounds analyzed in the present study to inhibit *Li*GDP-MP enzyme activity. Despite its lack of activity on the purified GDP-MP, compound−**11** displayed a reasonable antileishmanial activity on both axenic and intramacrophage amastigotes with IC_50_ at 13.3 µM and 9.5 µM, respectively, with a cytotoxicity at 32.6 µM (Table 1). These results show that the antileishmanial activity of **11** is not mediated through GDP-MP inhibition in the parasite.

Interestingly, miltefosine, a reference drug used in the treatment of leishmaniasis, did not show any activity on leishmanial GDP-MP, despite antileishmanial activities at the micromolar level (Table 1). Therefore, the mechanism of action of this alkylphosphocholine, with a saturated linear C_16_ chain (hexadecylphosphocholine), is not mediated by the inhibition of leishmanial GDP-MP. Moreover, these results show the importance of the resorcinol group at the end of the linear aliphatic chain of compounds **9** and **10** to inhibit GDP-MP.

The structures of compounds **4–11** are formed with a mono- or dihydroxyphenol ring and a long linear aliphatic carbon chain, which gives these compounds an amphiphilic character [16]. They have a strong affinity for biological membranes, resulting in significant changes in the biophysical properties of the lipid bilayers. [17]. Although the membrane disrupting effect is highly dependent on the condition in which they interact in the lipid layer [18,19], this property may explain at least in part their antiprotozoal, antimicrobial, and cytotoxic activity [17].

Among all the compounds evaluated, compounds **1**, **3**, **9,** and **10** displayed an activity on the leishmanial target as well as on axenic amastigotes, but not on intramacrophage parasites. As a comparison, a 2-substituted quinoline named compound−**99** was previously identified to inhibit specifically the leishmanial GDP-MP *Ld*GDP-MP with a K*_i_* at 7 µM and to display an IC_50_ at the micromolar level on both axenic and intramacrophage *L. donovani* amastigotes, but with a cytotoxicity in the same range [12]. Furthermore, compound−**100**, a 4-substituted quinoline, was previously shown to display an antileishmanial activity on both axenic and intramacrophage amastigotes of *L. donovani*, *L. mexicana*, and *L. major* IC_50s_ between 12 µM and 49 µM, and to inhibit both *Ld*GDPMP and *h*GDP-MP with a K*_i_* between 20 and 62 µM [11,12]. This compound was also able to inhibit *L. major* GDP-MP with an interesting IC_50_ at 0.58 µM, which is lower than the more active compounds reported in the present work [11]. As the activity of compounds **1**, **3**, **9,** and **10** is only moderate on axenic amastigotes, the synthesis of new derivatives could allow an improvement in their antileishmanial activity as well as their affinity and their specificity for the leishmanial GDP-MP. In the literature, some alkylresorcinols have been shown to display an antileishmanial activity at the micromolar to submicromolar level [20,21]. These alkylresorcinols have 17 carbons in their alkyl chain, as does compound **10** in our study, with at least two unsaturations. Therefore, some derivatives of compound **10** with several unsaturations in the alkyl chain would be useful to be further synthesized and evaluated for their antileishmanial activity as well as their capacity to inhibit the leishmanial GDP-MP.

Moreover, the absence of activity of compounds **1**, **3**, **9,** and **10** on intramacrophage parasites could be due to an inability of the compounds to cross the macrophage plasma membrane and/or the membrane of the parasitophorous vacuole before reaching the parasite. Therefore, a formulation of these compounds would increase their ability to reach the parasite in its vacuole, and thus would promote their antileishmanial activity on intramacrophage amastigotes. Among the possible formulations that could be used, a liposomal formulation may be the most relevant as the alkyl chain of the compounds could insert in liposomal lipid bilayers, and liposomes are targeted in vivo to the liver of infected animals where the parasites proliferate during visceral leishmaniasis.

## 3. Materials and Methods

### 3.1. Plant Materials

Wood of *Virotia francii* (Guill.) P.H.Weston & A.R.Mast (Proteaceae) and bark of *Tapeinosperma schlechteri* Mez (Primulaceae) were collected in New Caledonia (Prony bay in August 2005, and “Forêt faux bon secours” in May 1998, under the reference DUM-0542 and LIT-0517, respectively). Herbarium specimens were deposited at the Herbier IRD de Nouméa.

Bark and twigs of *Knema hookeriana* Warb. (Myristicaceae) were collected in April 1996 in Gunung Bujang Melaka (Kampar, Perak, Malaysia). A voucher specimen (KL-4584) was deposited in the herbarium of the Department of Chemistry of the Science Faculty, University of Malaya (Kuala Lumpur, Malaysia).

### 3.2. Extraction and Compounds Isolation

Air dried wood of *Virotia francii* (900 g), bark and twigs of *Knema hookeriana* (100 g) and bark of *Tapeinosperma schlechteri* (100 g) were extracted by maceration in EtOAc (3 × 2 L, 3 × 300 mL and 3 × 300 mL, respectively), to yield 15 g (CE-1), 2.5 g (CE-2) and 0.8 g (CE-3) of crude extracts, respectively, after concentration in vacuo at 40 °C. The crude extract CE-1 (7.7 g) was subjected to silica gel column flash chromatography (RediSep Teledyn Isco, 120 g) using a gradient of *n*-heptane-CH_2_Cl_2_ (50:50 to 0:100), then CH_2_Cl_2_-MeOH (100:0 to 80:20) to afford 17 fractions F1-F17. Fraction F14 (CH_2_Cl_2_-MeOH 90:10) was subjected to a preparative HPLC (Kromasil C_18_ column, 250 × 21.2 mm, 5 μm, at 21 mL/min) using an isocratic mobile phase of MeCN-H_2_O 87:13 + 0.1% formic acid to afford compounds **1–3** (19.6, 4.5 and 82.7 mg, respectively). The isolation of compounds **4–8** from CE-2 is described in reference [22]. The crude extract CE-3 was subjected to a preparative HPLC using an isocratic mobile phase of MeCN–H_2_O 85:15 + 0.1% formic acid to afford compounds **9–11** (39, 65, and 170 mg, respectively).

### 3.3. Structural Identification of Compounds (**1–11**, Figure 1)

NMR spectra were recorded with a Bruker spectrometer (Avance 300 MHz for ^1^H and 75 MHz for ^13^C, Bruker, Wissembourg, France) using CDCl_3_ as solvent. MS data were acquired using a Waters Acquity UPLC coupled to a Waters LCT Premier XE mass spectrometer (HRMS analysis) or to a Waters Acquity TQ Detector (MS2 analysis).

From *V. francii:* Oncostemonol D, (8′*Z*)-1-hydroxy-3-methoxy-5-[16′-(3′′,5′′-dihydroxy-phenyl)-8′-hexadecen-1′-yl]benzene (**1**) was isolated [23].

The structure of novel oncostemonols I and J (**2** and **3**) were elucidated on the basis of extensive 1D and 2D NMR spectroscopic interpretation. The structure of oncostemonols I (**2**) and J (**3**) were very similar to that of **1**, except for the absence of the Δ^8′,9′^ double bond for compound **2**, and the presence of an unsaturated alkyl chain including two additional methylene groups for compound **3**. The stereochemistry at the double bond between C-10′/C-11′ of **3** was assigned as *Z*, as in the case of oncostemonol D, on the basis of the close similarity of coupling constants and ^13^C NMR values [24]. The location of the double bonds on the carbon chains of compounds **1** and **3** were determined from ESIMS experiments in negative mode. In the MS spectrum of **3**, the mass fragments observed at 191, 233, 245, and 287 are indicative of a pattern of peaks with a 54 amu window, which is composed of peaks due to allylic cleavage via 1,4-elimination of H_2_ [25]. Combined with biogenetic consideration, it can deduced from these results that the double bond was located at C-10′ [26].

Oncostemonol I, 1-hydroxy-3-methoxy-5-[16′-(3′′,5′′-dihydroxyphenyl)-hexadecanyl]benzene (**2**). Colorless liquid, negative ESI-HR-MS, *m/z* 455.3157 [M − H]^−^ [calcd. for C_29_H_43_O_4_, 455.3156]. ^1^H NMR (300 MHz, CDCl_3_, δ, ppm, *J*/Hz): 1.29–1.58 (28H), 2.50 (4H), 3.72 (OCH_3_), 6.08, (1H, t, *J* = 2.1, H-4′′), 6.14 (2H, d, *J* = 2.0, H-2′′, H-6′′), 6.17 (1H, t, *J* = 2.1, H-2), 6.22 (2H, d, 2.0 Hz, H-4, H-6). ^13^C NMR (75 MHz, CDCl_3_, δ, ppm): 29.2–31.2 (C-2′-C-15′), 36.3 (C-1′, C16′), 55.6 (OCH_3_), 99.2 (C-2), 100.4 (C-4′′), 107.8 (C-2′′, C-6′′), 106.2 (C-4), 108.7 (C-6), 146.1 (C-5, C-1′′), 157.9 (C-3′′, C-5′′), 158.0 (C-1), 161.4 (C-3).

Oncostemonol J, (10′*Z*)-1-hydroxy-3-methoxy-5-[18′-(3′′,5′′-dihydroxy-phenyl)-10′-octodecen-1′-yl]benzene (**3**). Colorless liquid, negative ESI-HR-MS, *m/z* 481.3333 [M − H]^−^ [calcd. for C_31_H_45_O_4_, 481.3312]. ^1^H NMR (300 MHz, CDCl_3_, δ, ppm, *J*/Hz): 1.29–1.58 (12H, H-2′-H-7′, H-12′-H-17′), 2.01 (2H, H-8′, H-11′), 2.50 (2H, H-1′, H-18′), 3.75 (OCH_3_), 5.34 (2H, t, *J* = 4.7 Hz, H-9′, H-10′), 6.17, (1H, t, *J* = 2.1, H-4′′), 6.23 (1H, t, *J* = 2.1, H-2), 6.24 (2H, d, *J* = 2.0, H-2′′, H-6′′), 6.27 (1H, d, 2.0 Hz, H-6), 6.32 (1H, d, 2.0 Hz, H-4). ^13^C NMR (75 MHz, CDCl_3_, δ, ppm): 27.3–31.2 (C-2′-C-9′, C-12′-C-17′), 36.6 (C-1′, C18′), 55.6 (OCH_3_), 98.9 (C-2), 100.3, (C-4′′), 106.9 (C-4), 108.0 (C-6, C-2′′, C-6′′), 130.1 (C-10′, C-11′), 145.9 (C-1′′), 146.2 (C-5), 156.7 (C-3′′, C-5′′), 156.8 (C-1), 160.8 (C-3).

From *K. hookeriana*: Khookerianic acid B (**4**), anagigantic acid (**5**), (*Z*)-2-hydroxy-6-(tridec-8-en-1-yl)benzoic acid (**6**), 2-hydroxy-6-tridecylbenzoic acid (**7**), (*Z*)-2-hydroxy-6-(pentadec-10-en-1-yl)benzoic acid (**8**) [22].

From *T. schlechteri*: (8′*Z*)-5-(pentadeca-8′-enyl)benzene-1,3-diol (**9**) [27], (8′*Z*)-5-(heptadeca-8′-enyl)benzene-1,3-diol (**10**) [27], and (8′*Z*)-1-hydroxy-3-methoxy-5 (pentadeca-8′enyl)benzene (**11**) [28].

### 3.4. GDP-MP Production and Purification

Recombinant His6 tagged GDP-MP from *L. infantum* (*Li*GDP-MP) was produced in *E. coli* and purified using Ni-nitrilotriacetic acid (Ni-NTA) and size exclusion chromatography Hiload^TM^16/60 Superdex^TM^ 200 column as previously described for *Ld*GDP-MP [12]. Recombinant His-tagged GDP-MP from human (*h*GDP-MP) was produced in *E. coli* and purified using a Mono-Q anion exchange column followed by a Hiload^TM^ 16/60 Superdex^TM^ 200 column as previously described [12].

### 3.5. Enzyme Assays

GDP-MP catalyzes the following reaction:

Man-1-P + GTP → GDP-Mannose + Pyrophosphate

Enzyme activities of both *Li*GDP-MP and *h*GDP-MP were measured at 37 °C for 20 min in 384 well plates in a 20-µL reaction containing 50 mM Tris-HCl pH 7.5, 5 mM MgCl_2_, 100 µM Mannose-1-phosphate, 100 µM GTP, 1 mM DTT, 0.1 U/mL inorganic pyrophosphatase (Sigma-Aldrich, Saint-Quentin-Fallavier, France) and 2 ng/µL of recombinant GDP-MP [12,29]. The reaction was initiated by the addition of GDP-MP, and was stopped after adding 20 µL of revelation buffer containing malachite green (0.03% *w/v*), ammonium molybdate (0.2% *w/v*) and Triton X-100 (0.05% *v/v*) in HCl (0.7 M) for 5 min at 37 °C. The quantity of inorganic phosphate generated, representing GDP-MP activity, was determined by measuring OD_650nm_ with a spectrometer plate reader Polar Omega (BMG LabTech, Champigny-sur-Marne, France). The molar extinction coefficient ε of the end product detected was determined at 27,207 M^−1^cm^−1^ as previously described [12].

### 3.6. Evaluation of Compounds on Purified Enzymes

To evaluate compounds on purified GDP-MPs, enzyme activities were measured as described above in the presence of 100 µg/mL of natural products originating from a chemical library from ICSN (Institut de Chimie des Substances Naturelles, Gif-sur-Yvette). The inhibitors were added in the reaction mix, before the addition of GDP-MP. The percentage of inhibition was then calculated in reference to untreated enzyme. The statistical parameter (Z-factor) was calculated to validate the quality of the HTS assays as described previously [30]. As a control, compounds were evaluated in parallel in the same conditions on inorganic pyrophosphatase alone in order to discard false-positives. No significant inhibition (<5%) of inorganic pyrophosphatase was observed with the positive compounds selected in the present study. Furthermore, no effect was observed on the activity of both *Li*GDP-MP and *h*GDP-MP with 1% DMSO, corresponding to the maximal solvent concentration used at 100 µg/mL of compound. Compounds presenting a minimum of 50% inhibition on a leishmanial GDP-MP were subsequently selected to determine, in a separate experiment, their IC_50_ by using two-fold dilutions of the compounds from 100 µg/mL to 3 ng/mL. The IC_50_ were determined by nonlinear regression using GraphPad Prism 7.0 and further converted in µM in order to normalize units in Table 1. Enzyme inhibition assays were performed in triplicate in three independent experiments.

### 3.7. Cell Cultures

Promastigotes of *Leishmania infantum* (MHOM/FR/2008/LEM5700) were cultured in the dark at 26 °C with 5% CO_2_ in M199 complete medium containing M199 medium supplemented with adenosine (100 µM), hemin (0.5 mg/L), Hepes (40 mM) pH 7.4, and heat inactivated foetal bovine serum (10%; HIFBS). Cultures of axenic amastigotes of *L. infantum* were adapted from [31]. Briefly, axenic amastigotes of *L. infantum* were obtained from late log promastigotes diluted at 1 × 10^6^/mL in M199 complete medium acidified at pH 5.5 and cultured at 37 °C with 5% CO_2_.

The RAW 264.7 macrophages (ATCC) were cultured at 37 °C with 5% CO_2_ in DMEM complete medium containing Dulbecco′s Modified Eagle′s Medium (DMEM, Invitrogen, Thermo-Fisher, Villebon-sur-Yvette, France) supplemented with penicillin-streptomycin (100 U/mL; Invitrogen), and heat-inactivated fetal bovine serum (10%; HIFBS).

### 3.8. In Vitro Evaluation of Compounds’ Cytotoxicity

Cytotoxicity was evaluated on RAW 264.7 macrophages. Cells were plated in 96-well microplates at a density of 2 × 10^4^ cells per well. After an incubation of 24 h at 37 °C with 5% CO_2_, the medium was removed in each well, and 100 µL of DMEM complete medium containing two-fold serial dilutions of the compounds, from 100 µM to 0.049 µM, was added to each well. After 48 h of incubation at 37 °C with 5% CO_2_, 10 µL of resazurin (450 µM) was added to each well, and further incubated in the dark for 4 h at 37 °C with 5% CO_2_. In living cells, resazurin is reduced in resorufin, and this conversion is monitored by measuring OD_570nm_ (resorufin) and OD_600nm_ (resazurin; multimode microplate reader Spark^®^, Tecan, Lyon, France). The cytotoxicity of the compounds was expressed as CC_50_ (Cytotoxic Concentration 50%: concentration inhibiting macrophage metabolic activity by 50%) and was determined by nonlinear regression in GraphPad Prism 7.0. In vitro cytotoxicity assays were performed in triplicate in three independent experiments.

### 3.9. In Vitro Antileishmanial Evaluation of Compounds on Axenic and Intramacrophage Amastigotes

The evaluations of activity on axenic amastigotes were adapted from the protocols previously described [12]. Briefly, two-fold serial dilutions of the compounds, from 100 µM to 0.049 µM, were performed in 100 µL of complete medium (see above) in 96-well microplates. Axenic amastigotes were then added to each well at a density of 10^6^/mL in a 200 µL final volume. After 72 h of incubation at 37 °C with 5% CO_2_, 20 µL of resazurin (450 µM) was added to each well and further incubated in the dark for 24 h at 37 °C with 5% CO_2_. Cell viability was then monitored as described above. The activity of the compounds was expressed as IC_50_, which was determined by nonlinear regression using GraphPad Prism 7.0. Miltefosine was used as the reference drug.

Concerning the evaluation on intramacrophage amastigotes, the determination of the cytotoxicity as presented above was necessary to use the highest drug concentrations to be studied on the intramacrophage amastigote model. RAW 264.7 macrophages were plated in 16-well Lab-Tek chamber slides (Thermo-FisherVillebon-sur-Yvette, France) at a density of 2 × 10^4^ cells per well and incubated for 24 h at 37 °C with 5% CO_2_. Axenic amastigotes were differentiated as described above, centrifuged at 2000× *g* for 10 min, resuspended in DMEM complete medium, and added to each well to reach a 16:1 parasite to macrophage ratio. After 24 h of infection at 37 °C with 5% CO_2_, extracellular parasites were removed, and DMEM complete medium (100 µL) containing two-fold serial dilutions of the compounds, from 100 µM to 0.049 µM, was added to each well. A positive control treated with 1% DMSO was added to each Lab-tek chamber-slide. After 48 h of treatment, the medium was removed and cells were fixed in methanol for 1 min, stained in 10% Giemsa (Merck) for 5 min, and further rinsed in water before observation in phase contrast at the microscope (Olympus CX31; Olympus, Rungis, France). The number of amastigotes was counted by two independent experimenters for a total of 300 macrophages per well and the ratio of amastigotes per macrophage was determined for each condition. This ratio was further compared with the one of the positive control, considered as 100%, to determine a percentage of inhibition (% I), as follows: % I = 100 − ((ratio of amastigotes per macrophage in treated cells/ratio of amastigotes per macrophages in untreated cells) × 100). The activity of the compounds was expressed as IC_50_, which was determined by nonlinear regression using GraphPad Prism 7.0. Miltefosine was used as the reference drug. In vitro antileishmanial evaluations were performed in triplicate in three independent experiments.

## Figures and Tables

**Table 1:** Evaluation of resorcinol derivatives on *Li*GDP-MP, *h*GDP-MP, and on *L. infantum* axenic and intramacrophage amastigotes, and their cytotoxicity.

Name	*Li*GDP-MP IC_50_ (µM*)* ± SD	*h*GDP-MP IC_50_ (µM) ± SD	*L. infantum* Axenic Amastigotes IC_50_ (µM) ± SD	*L. infantum* Intramacrophage Amastigotes IC_50_ (µM) ± SD	Cytotoxicity on RAW 264.7 CC_50_ (µM) ± SD	Selectivity Index
**1** **Oncostemonol D**	3.5 ± 11.4	1.3 ± 0.2	78.9 ± 3.1	>50	34.4 ± 1.5	<0.7
**2** **Oncostemonol I**	5.9 ± 3.5	13.7 ± 14.2	ND	ND	ND	ND
**3** **Oncostemonol J**	2.3 ± 8.1	1.9 ± 0.4	49.5 ± 4.2	>25	31.8 ± 1.6	<1.2
**4** **Khookerianic acid B**	120.0 ± 27.5	79.9 ± 5.9	>100	>100	83.1 ± 7.0	<0.8
**5** **Anagigantic acid**	50.6 ± 4.8	38.3 ± 4.1	>100	>100	>100	/
**6**	100.2 ± 16.6	122.2 ± 26.1	>100	42.7 ± 1.3	>100	>2.3
**7**	16.9 ± 5.0	29.0 ± 2.5	>100	>100	>100	/
**8**	9.8 ± 3.2	13.9 ± 0.6	>100	>100	>100	/
**9**	11.0 ± 4.7	31.1 ± 10.7	53.3 ± 4.6	>25	26.1 ± 6.4	<1
**10**	14.1 ± 3.8	21.9 ± 6.3	25.9 ± 5.9	>25	17.9 ± 1.3	<0.7
**11**	>300	ND	13.3 ± 4.4	9.5 ± 2.1	32.6 ± 7.4	3.4
**Miltefosine**	>250	ND	1.0 ± 0.3	6.7 ± 1.7	54.2 ± 5.8	8.1

Selectivity Index = CC50/IC50 on intramacrophage amastigotes, ND = Not Determined.

## Data Availability

All data are already provided in the manuscript.

## Sample Availability

Samples of the compounds are available from the authors.
